# Treatment of idiopathic scoliosis with conservative methods based on exercises: a systematic review and meta-analysis

**DOI:** 10.3389/fspor.2024.1492241

**Published:** 2024-12-23

**Authors:** Vanja Dimitrijević, Bojan Rašković, Miroslav Popović, Dejan Viduka, Siniša Nikolić, Patrik Drid, Borislav Obradović

**Affiliations:** ^1^ Faculty of Sports and Physical Education, University of Novi Sad, Novi Sad, Serbia; ^2^ Technical Faculty, Singidunum University, Belgrade, Serbia; ^3^ Faculty of Applied Management, Economics, and Finance in Belgrade, University of Business Academy, Novi Sad, Serbia; ^4^ Department of Paediatric Rehabilitation, Dr Miroslav Zotovic Institute for Physical Medicine and Rehabilitation, Banja Luka, Bosnia and Herzegovina; ^5^ Department of Physiotherapy, Faculty of Medicine, University of Banja Luka, Banja Luka, Bosnia and Herzegovina

**Keywords:** conservative methods, idiopathic scoliosis, Cobb angle, ATR, meta-analysis

## Abstract

**Introduction:**

This systematic review and meta-analysis aimed to systematically assess the effect size of conservative methods based on exercise for respondents with idiopathic scoliosis.

**Methods:**

This study was developed in accordance with the PRISMA guidelines. The PubMed, Cochrane Library, Web of Science, and Google Scholar databases were searched in May 2023. The key search terms were “Idiopathic scoliosis”, “Adolescent idiopathic scoliosis”, “Cobb angle”, “Angle of trunk rotation”, “Quality of life”, “Schroth method”, and “Core stabilization exercises”. Risk of bias was assessed for each randomized trial using the Cochrane risk of bias tool, and the methodological index for non-randomized studies. The outcomes included Cobb angle, angle of trunk rotation (ATR), forced vital capacity (FVC), forced expiratory volume in 1 s (FEV1), and quality of life (QoL). R 4.0.5 software was used, and standardized mean differences (SMD) and 95% confidence intervals (CIs) were calculated for continuous outcomes using a random model.

**Results:**

In total, 23 studies were included. Depending on the outcome measured, the effect size of the different methods in the treatment of idiopathic scoliosis ranged from small to large as follows: Cobb angle (SMD = −0.43, *p* < 0.0001), ATR (SMD = −0.25, *p* = 0.06), FVC (SMD = 0.48, *p* = 0.03), FEV1 (SMD = 0.51, *p* = 0.004), and QoL (SMD = 0.95, *p* < 0.0001).

**Conclusion:**

Our meta-analysis indicates the positive effects of applying conservative methods based on therapeutic exercises on patients with idiopathic scoliosis.

**Systematic Review Registration:**

https://www.crd.york.ac.uk/prospero/display_record.php?RecordID=373554, PROSPERO (CRD42022373554).

## Introduction

Idiopathic scoliosis (IS) is a complex three-dimensional (3D) deformity of the spine and can develop in healthy children at any stage of growth (1, 2). IS is classified according to the age of the patient at the time of diagnosis (3). The condition is divided into three types: infantile scoliosis (under 3 years old), juvenile scoliosis (3–9 years old), and adolescent scoliosis (10–18 years old) (4). The adolescent form accounts for most cases of idiopathic scoliosis (5). Curvature in the spine can develop at any level of the non-cervical spine, and depending on the vertebrae that are affected, it is called thoracic, thoracolumbar, or lumbar scoliosis (6). When a diagnosis of scoliosis is made, the primary concern is whether there is an underlying cause and whether the curve will progress. The three main determinants of progression are the gender of the patient, future growth potential, and the size of the curve at the time of diagnosis (7).

There are surgical and non-surgical (conservative) treatment methods in the treatment of IS. There is consensus on surgical treatment in the minority of patients with spinal curvature greater than 45°, particularly in patients with severe rotational abnormalities (8). The vast majority of adolescents with idiopathic scoliosis receive conservative treatments. The most common interventions used in the conservative treatment of adolescent idiopathic scoliosis (AIS) are bracing, and exercise therapy (8, 9). Conservative therapies such as physiotherapy scoliosis-specific exercises (PSSEs), with or without simultaneous external bracing, are used as an alternative for patients who have a curvature below 50° (10). Some studies using PSSEs have shown improvement in neuromotor control (11, 12), respiratory functions (13), back muscle strength, and cosmetic appearance (13). The Scientific Exercise Approach to Scoliosis (SEAS) is a scoliosis treatment method that focuses on restoring postural control and improving stability through exercises that include active 3D self-correction of scoliotic posture. Active 3D self-correction is achieved first through patient education and increasing patient awareness of their deformity (14). The largest number of studies that provide quantitative results focus on therapies that use therapeutic exercise as a form of treatment for idiopathic scoliosis, and the Schroth method and core stabilization exercises are the most often used treatments in these studies (15, 16).

Previous research confirms that these methods have positive effects in reducing the Cobb angle (13, 17–19). A reduction in the Cobb angle is also followed by a decrease in the angle of trunk rotation (ATR) (20–22), and patients’ subjective condition, as assessed through Scoliosis Research Society (SRS) questionnaires, showed significant improvement (22–24) Some studies (25–27) also confirm that pulmonary function results significantly improve with the application of these methods. Previous meta-analyses (15, 16) confirm the positive effects of the Schroth method and core stabilization exercises in treating idiopathic scoliosis.

The reason for the increasing use of non-surgical methods is that surgical procedures carry significant risks. Neurological complications can lead to nerve root damage, spinal cord deficit, extrinsic spinal compression, epidural hematoma or abscess, nerve element injury, or spinal cord distraction during correction (28). Reported risk factors for neurological complications during AIS surgery include a Cobb angle greater than 90°, kyphosis correction, vertebral osteotomies, combined anterior and posterior fusions, and reoperation (29, 30). There may also be delayed neurological complications caused by progressive ischemia of the spinal cord resulting in the development of an epidural hematoma (31). The reported prevalence of non-neurological complications after AIS surgery ranges from 0% to 30% (30, 32–34). These are only a few of the reasons that lead patients to turn more and more to conservative (non-surgical) treatment methods, and researchers to better quantify the effects of such methods. For this reason, our previously mentioned research studies related to idiopathic scoliosis and other problems related to the spine (35, 36) are based on conservative (non-surgical) treatment.

Despite promising evidence, there are significant limitations in the current literature, such as small sample sizes, heterogeneity in exercise protocols, and a low number of outcomes in studies. These limitations make it difficult to establish a standardized, evidence-based approach for idiopathic scoliosis. A systematic review and meta-analysis are necessary to address these gaps by synthesizing existing data to provide a comprehensive assessment of treatment efficacy. This approach will enable clearer conclusions on optimal exercise protocols, offer insight into long-term effectiveness, and support clinical decision-making by consolidating diverse findings into a more robust evidence base. This research aimed to find and unify the results of as many studies as possible that met the conditions for conservative methods based on therapeutic exercises, and to use a meta-analysis to show the effects of their application on different outcomes in subjects with IS.

## Methods

### Study design

This study was developed following the PRISMA guidelines (37), and Cochrane Collaboration guide (38).

### Data sources and search strategy

**Figure 1:**
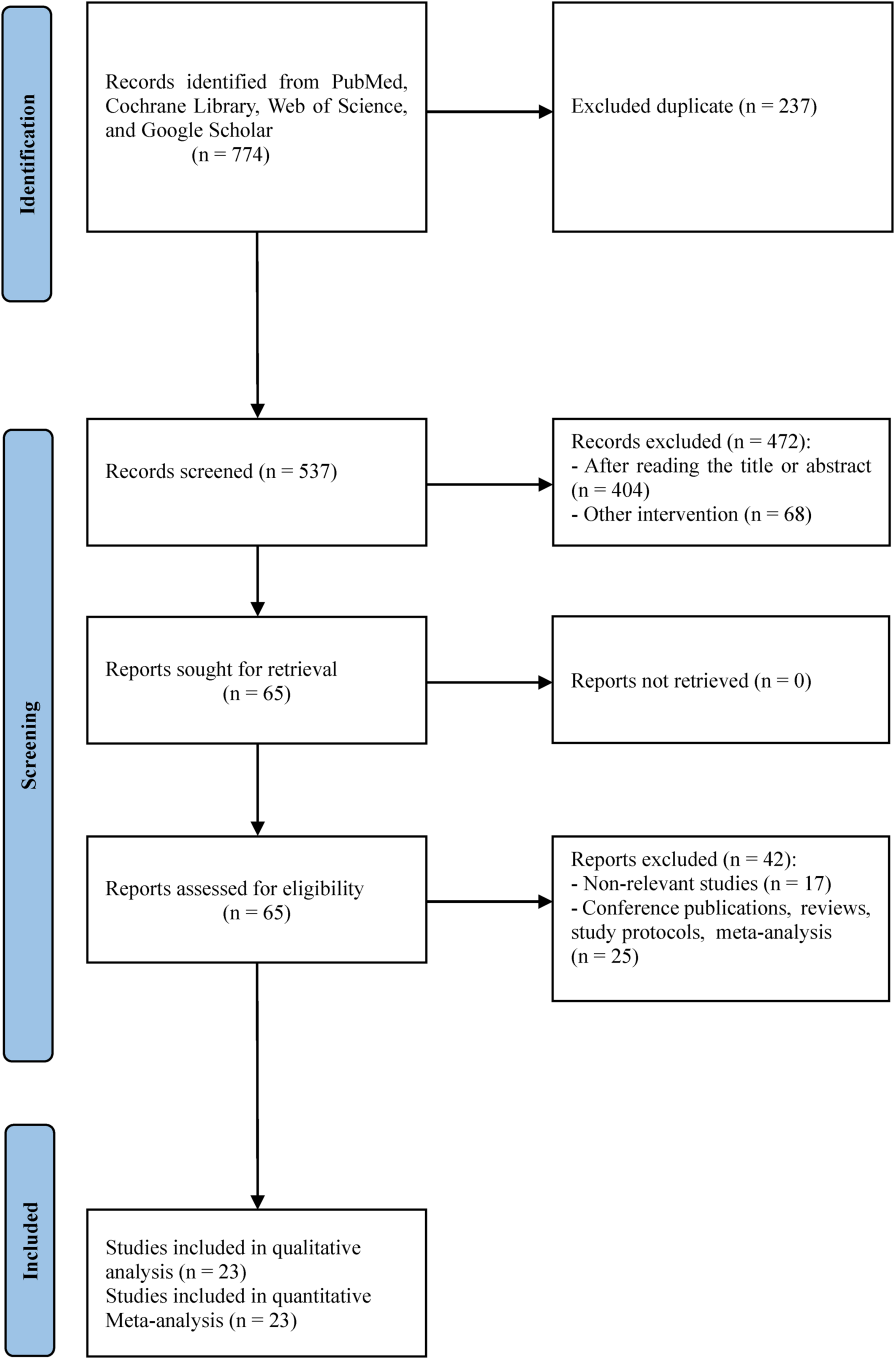
Flow chart.

The search was developed by identifying relevant studies that used different conservative methods in the treatment of IS. The search included the following databases: PubMed, Cochrane Library, Web of Science, and Google Scholar. The search was carried out in May 2023. Keywords used in the search are “Scoliosis” OR “Idiopathic scoliosis” OR “Adolescent idiopathic scoliosis” AND “Cobb angle” OR “Angle of trunk rotation” OR “Quality of life” AND “Physiotherapeutic Scoliosis Specific Exercises” OR “PSSE” OR “Schroth method” OR “Core stabilization exercises” OR “Pilates” OR “Exercise therapy” OR “Physical therapy” OR “Corrective exercise”. The search diagram is shown in Figure 1.

### Study selection

All studies had to meet PICOS (Population, Interventions, Comparators, Outcomes, Study Designs) criteria (39). P (population): diagnosed subjects with idiopathic scoliosis; I (intervention): different conservative methods based on exercises; C (comparison): control group received no treatment or received some other conservative treatment based on exercise; O (outcome): Cobb angle, angle of trunk rotation, forced vital capacity (FVC), forced expiratory volume in 1 s (FEV1), and quality of life (QoL).; S (study design): comparative studies published after 2000. There was no language restriction. Studies excluded were systematic reviews, meta-analyses, study protocols, books, book reviews, and conference publications. The process selection of studies was performed by two researchers (BO and DV), and decisions were made with consultation and consensus.

### Data extraction and quality assessment

After selecting studies based on all inclusion and exclusion criteria in the meta-analysis, two investigators independently performed data extraction. The table contains the following data: authors, year of publication, program type, number of participants, age, Cobb angle, outcomes, exercise time, and duration.

The quality of the studies was independently assessed by two investigators (BR and VD). For randomized studies, the Cochrane risk of bias tool (38) was used. Each study was assessed as low risk, unclear risk, or high risk. To ascertain the quality of non-randomized studies, the methodological index for non-randomized studies (MINORS) was used (40).

### Data synthesis and analysis

R 4.0.5 software with the “meta” package was used. The effect size was estimated for each of the outcomes. For each study, standardized mean difference (SMD) and 95% confidence intervals (CIs) were calculated for continuous outcomes using a random model. Although the same measurement scales were used for all the outcomes in our study and the same method was used to determine the scoliotic curve via the Cobb angle, we used SMD due to possible differences in the use of different devices in radiography, which may have different specifications and parameters. Furthermore, differences could also occur due to the accuracy and consistency of body position, which is crucial for obtaining accurate and reliable radiographs, since even small deviations can lead to changes in the appearance of anatomical structures, which can affect the accuracy of the diagnosis. Spirometers from different manufacturers may have differences in sensitivity, measurement accuracy, and calibration, which can affect the results. Thus, we used SMD for these reasons. According to Cohen's guide, values of ≥0.2, ≥0.5, and ≥0.8 indicate small, medium, and large effect sizes, respectively (41). *p* <0.05 was considered statistically significant. Five subgroup analyses were performed for the Cobb angle outcome. Based on the reported treatment durations in the included studies, our assessment was that three subgroups could be formed. A subgroup analysis was performed for the duration of treatment factor for the Cobb angle outcome and three subgroups were formed: 8–10, 12, and >12 weeks. Subgroup analysis was performed for factor risk of bias and three subgroups were formed: low, moderate, and high. For the factor method, three subgroups were formed: Schroth exercise, core stabilization, and combined therapy, which included studies that used some other exercises or combined several types of exercises. Since some studies had respondents older than 18 years, we performed a subgroup analysis for the age factor and formed two subgroups: the younger group and the older group. A subgroup analysis was also performed in relation to whether the control group had treatment or not, and two subgroups were formed: treatment and non-treatment. A sensitivity analysis was also performed to observe how each study affected the overall effect size for the Cobb angle outcome. An analysis was also performed within the experimental and control groups, and within the control group, a subgroup analysis was performed, depending on whether the control group did or did not have treatment. Higgins’ *I*2 test was used to assess heterogeneity. Egger's test evaluated the publication bias for the Cobb angle outcome.

## Results

### Study selection and characteristics

Initially, 774 studies were selected from the four databases and 237 studies were immediately excluded because they were duplicates. Thus, 537 studies were selected for further analysis. After screening the titles and abstracts, 65 studies were screened in full. Of these, 42 studies were excluded and the remaining 23 studies were included in the qualitative and quantitative analysis. The study selection flow process is presented in Figure 1. Table 1 shows some of the characteristics of the studies we included. A total of 796 respondents participated in the 23 studies. In the selected studies, the sample size was between 15 and 110 subjects. The treatment lasted from 6 weeks to 6 months.

**Table 1:** Description of the included studies.

Study	*N*	Program type	Outcome	Cobb angle	Age	Exercise time	Duration
Alayat et al. (42)	50	Direction-sensitive exercise therapy vs. traditional exercises therapy	Cobb angle	13.20° ± 4.1°	14.1 ± 1.6	N/A	12 weeks
				12.68° ± 3.7°	14.4 ± 1.7		
Duangkeaw et al. (20)	16	Schroth 3D exercise vs. Kinesio tape with Schroth exercise	ATR	N/A	10–18	120 min	6 weeks
			Inspiratory muscles strength				
			Expiratory muscles strength				
			Muscle endurance of back				
			General mobility				
Gür et al. (43)	25	Core stabilization vs. control (traditional rehabilitation)	Cobb angle	N/A	14.2 ± 1.8	N/A	10 weeks
			Rotation		14 ± 1.6		
			QOL				
HwangBo (44)	16	Schroth exercise vs. Pilates exercise	Cobb angle	22.07° ± 6.81°	18.14 ± 1.6	N/A	12 weeks
			Psychological factors	21.2° ± 3.95°	18.88 ± 1.55		
HwangBo (21)	16	Schroth exercise group	Cobb angle	18.98° ± 0.03°°	20.94 ± 0.32	60 min	12 weeks
		Pilates exercise group	ATR	19.02° ± 9.01°°	21.08 ± 1.95		
			Chest expansion				
Kim et al. (45)	40	Swiss ball exercise	FVC, FEV1, FEV1/FVC, TIS	N/A	18.5 ± 1.2	30 min	8 weeks
		Resistance exercise			17.9 ± 1.1		
Kim and Hwangbo (46)	24	Schroth exercise vs. Pilates exercise	Cobb angle	23.63° ± 1.5°	15.6 ± 1.1	60 min	12 weeks
			Weight distribution	24° ± 2.6°	15.3 ± 0.8		
Kim and Park (17)	15	SERME	Cobb angle	24.49° ± 8.32°	17.75 ± 4.71	60 min	8 weeks
		Schroth 3D exercise	Pulmonary function	27.16° ± 12.44°	15.57 ± 2.70		
			Functional movement screen				
Ko and Kang (18)	29	Core stabilization vs. control (no treatment)	Cobb angle	10°–20°	12.71 ± 0.72	60 min	12 weeks
			Flexibility		12.8 ± 0.86		
			Lumbar flexion muscle				
			Lumbar extension muscle				
Kocaman et al. (22)	28	Schroth exercise vs. core exercise	Cobb angle	10°–30°	14.07 ± 2.37	60 min	10 weeks
			ATR		14.21 ± 2.19		
			Cosmetic trunk deformity				
			Spinal mobility				
			QOL				
Kumar et al. (26)	36	Oriented ergonomics exercises	Cobb angle	12.61° ± 1.81°	12.17 ± 1.72	40 min	12 months
		Spinal strengthening exercises	FVC, FEV1, FEV1/FVC, PEF, VC	12.72° ± 1.40°	11.56 ± 1.46		
Kuru et al. (47)	30	Schroth 3D exercise vs. Control (no treatment)	Cobb angle	10°–60°	10–18	90 min	6 weeks
			ATR				3 months
			Asymmetry				6 months
			Quality of life				
Langensiepen et al. (48)	38	Scoliosis-specific exercises + WBV vs. Control (scoliosis-specific exercises)	Cobb angle	30.1° ± 9.0°	13.6 ± 1.6	N/A	6 months
				29.65° ± 8.7°	14.0 ± 0.9		
		
Lee and Lee (49)	15	Schroth exercise vs. control (no treatment)	Cobb angle	22.11° ± 7.58°	18.88 ± 3.06	120 min	12 weeks
			Vertebral rotation angle	22.17° ± 7.27°	24.14 ± 12.69		
			Weight bearing in feet				
			Rotation volume				
Mohamed and Yousef (50)	34	Schroth exercise	Cobb angle	20.42° ± 2.57°	14.50 ± 1.20	60 min	6 months
		Proprioceptive neuromuscular facilitation	ATR	20.21° ± 2.80°	14.90 ± 1.40		
			Plantar Pressure Distribution				
			Functional Capacity				
Monticone et al. (24)	110	Task-oriented spinal exercises and vs. control (traditional spinal exercises)	Cobb angle	19.3° ± 3.9°	12.5 ± 1.1	60 min +	N/A
			QOL	19.2° ± 2.5°	12.4 ± 1.1	30-min in home	Follow-up
							12 months
Noh et al. (51)	32	Corrective spinal technique vs. control (conventional exercise)	Cobb angle	21.6° ± 10.1°	13.8 ± 2.8	60 min	4 months
			Vertebral rotation	19° ± 7°	14.9 ± 2.3		
			Thoracic kyphosis				
			Lumbar lordosis				
			Pelvic tilt				
			Pelvic incidence				
Park et al. (52)	51	Core strengthening vs. home program	Cobb angle	10°–20°	20 ± 2	50 min	10 weeks
			Muscle strength		20.6 ± 1.8		
Park et al. (53)	33	Core stabilization vs. control (manual massage)	Cobb angle	15.76° ± 2.72°	≥20	50 min	8 weeks
			Balance	17.81° ± 2.99°	14 ± 1.3		
Qi et al. (27)	38	Core stabilization vs. control (no treatment)	Cobb angle	24.06° ± 3.84°	13.94 ± 1.30	60 min	12 weeks
			FVC, FEV1, FEV1/FVC,	23.88° ± 2.37°	13.61 ± 1.33		
			MIP, MEP				
Schreiber et al. (23)	50	Schroth exercise	Quality of life	10°–45°	10–18	60 min	3 months
		Standard of care	Back extensor strength				6 months
Schreiber et al. (19)	50	Schroth exercise	Cobb angle	10°–45°	10–18	60 min	6 months
		Standard of care	Sum of curves				
Won et al. (54)	20	Neuromuscular stabilization technique vs. home exercise program	Cobb angle	16.56° ± 2.50°	14.50 ± 2.50	30 min	6 months
				18.90° ± 5.24°	15.90 ± 2.69		

N, number of subjects in the group; ATR, angle of trunk rotation; QOL, quality of life; ATI, angle of trunk inclination; VC, vital capacity; FVC, forced vital capacity; FEV1, forced expiratory volume in 1 s; MIP, maximum inspiratory pressure; MEP, maximum expiratory pressure; SERME, Schroth's three-dimensional exercises in combination with respiratory muscle exercise; WBV, whole body vibration; N/A, no answer; PEF, peak expiratory flow; VC, vital capacity; TIS, trunk impairment scale.

### Risk of bias

**Figure 2:**
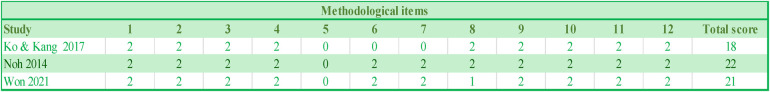
Risk of bias—non-randomized studies.

**Figure 3:**
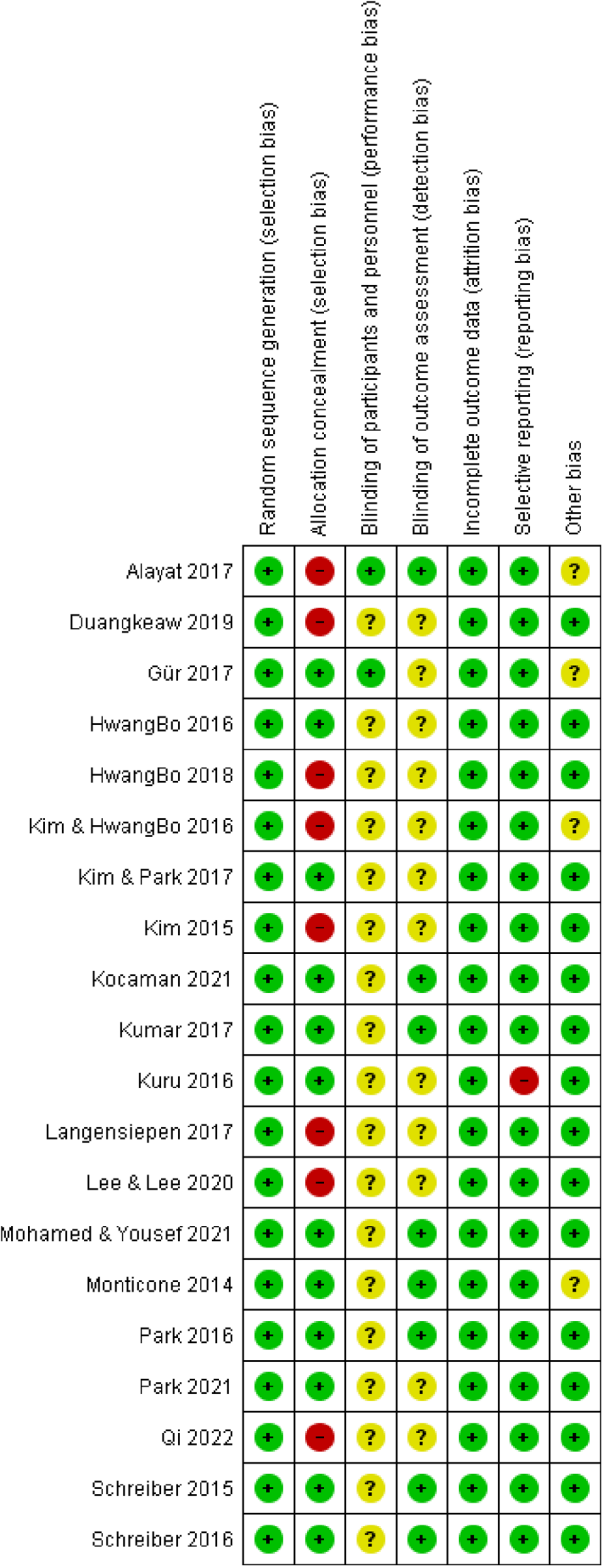
Risk of bias—randomized studies.

Figures 2, 3 show the risk of bias for the non-randomized and randomized studies, respectively. Of the 23 included studies, 3 were non-randomized, while 20 were randomized. Of the randomized studies, eight had a high risk in the item “Concealment of allocation” to the group. Participants and physiotherapists could not be blinded to the treatment due to the very nature of the treatment, although some studies (42, 43) report blinding in this item, while eight studies report blinding in the item “Blinding of outcome assessment”. A study (47) presented the data as median (min-max), which represents a problem for data processing, and this study was assessed as high risk in the item “Selective reporting”. Out of a total of 140 items, there were 97 (69.3%) low-risk items, 34 (24.3%) moderate-risk items, and 9 (6.4%) high-risk items. To ascertain whether the quality of the studies affected the effect size, we performed a subgroup analysis for the risk of bias for the Cobb angle outcome and presented the results in the results of the meta-analysis. All three non-randomized studies were comparative and had a minimum score of 18 and a maximum score of 22 out of a possible 24. In the subgroup analysis, two studies (51, 54) were in the moderate-risk subgroup, while one study (18) was in the high-risk subgroup.

### Cobb angle

**Figure 4:**
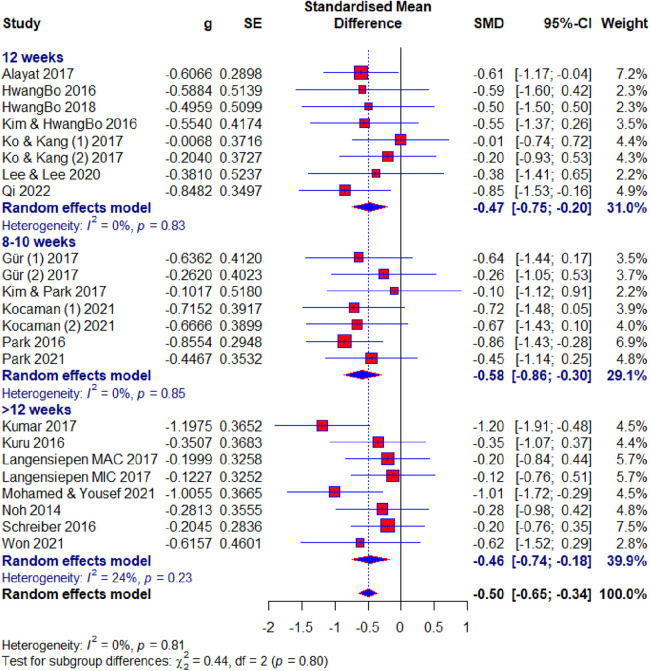
Forest plot—Cobb angle outcome: duration subgroups. MAC, major curve; MIC, minor curve.

**Figure 5:**
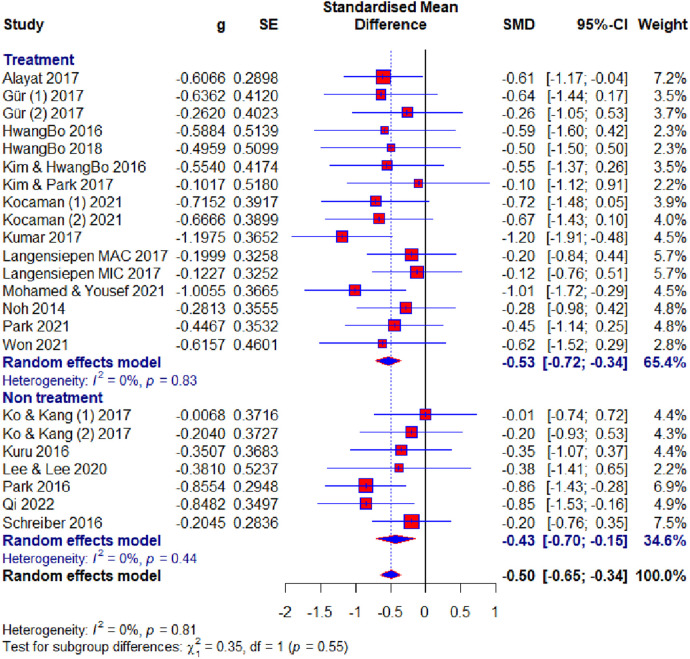
Forest plot—Cobb angle outcome: treatment subgroups.

Of the 23 included studies, 19 used the Cobb angle as an outcome. After the analysis, statistical significance was found (SMD = −0.50; 95% CI = −0.65 to −0.34; *p* < 0.0001) and there was no heterogeneity (*I*2 = 0%, *p* = 0.81). Subgroup analysis for the duration factor showed the following results: 8–10 weeks subgroup : (SMD = −0.58; 95% CI = −0.86 to −0.30; *p* < 0.0001; heterogeneity *I*2 = 0%, *p* = 0.85); 12 weeks subgroup: (SMD = −0.47; 95% CI = −0.75 to −0.20; *p* < 0.0001; heterogeneity *I*^2^ = 0%, *p* = 0.83); >12 weeks subgroup: (SMD = −0.46; 95% CI = −0.65 to −0.34; *p* < 0.0001; heterogeneity *I*^2^ = 24%, *p* = 0.23) (Figure 4). Subgroup analysis for the age factor showed the following results: younger subgroup: (SMD = −0.48; 95% CI = −0.65 to −0.31; *p* < 0.0001; heterogeneity *I*^2^ = 0%, *p* = 0.61); older subgroup: (SMD = −0.56; 95% CI = −0.89 to −0.23; *p* < 0.0001; heterogeneity *I*^2^ = 0%, *p* = 0.85). Subgroup analysis for the risk of bias showed the following results: low subgroup: (SMD = −0.62; 95% CI = −0.82 to −0.41; *p* < 0.0001; heterogeneity *I*^2^ = 0%, *p* = 0.67); moderate subgroup: (SMD = −0.38; 95% CI = −0.65 to −0.12; *p* < 0.0001; heterogeneity *I*^2^ = 0%, *p* = 0.87); high subgroup : (SMD = −0.23; 95% CI = −0.67 to −0.20; *p* < 0.0001; heterogeneity *I*^2^ = 0%, *p* = 0.62). Subgroup analysis for the different exercise methods showed the following results: combined therapy subgroup: (SMD = −0.45; 95% CI = −0.74 to −0.15; *p* < 0.0001; heterogeneity *I*^2^ = 13%, *p* = 0.33); core stabilization subgroup: (SMD = −0.50; 95% CI = −0.77 to −0.24; *p* < 0.0001; heterogeneity *I*^2^ = 0%, *p* = 0.50); Schroth exercise subgroup: (SMD = −0.53; 95% CI = −0.79 to −0.27; *p* < 0.0001; heterogeneity *I*^2^ = 0%, *p* = 0.88). Subgroup analysis for treatment usage in the control group showed the following results: treatment subgroup: (SMD = −0.53; 95% CI = −0.72 to −0.34; *p* < 0.0001; heterogeneity *I*^2^ = 0%, *p* = 0.83); non-treatment subgroup: (SMD = −0.43; 95% CI = −0.70 to −0.15; *p* < 0.0001; heterogeneity *I*^2^ = 0%, *p* = 0.44) (Figure 5). Analysis within groups showed the following results: experimental group: (SMD = 1.13; 95% CI = 0.69–1.57; *p* < 0.0001; heterogeneity *I*^2^ = 83%, *p* < 0.01) (Supplementary Material, Figure 6); control group: (SMD = 0.45; 95% CI = 0.18 to −0.71; *p* < 0.0001; heterogeneity *I*^2^ = 65%, *p* *<* 0.01). Subgroup analysis for treatment usage in the control group showed the following results: treatment subgroup: (SMD = 0.62; 95% CI = 0.28–0.96; *p* < 0.0001; heterogeneity *I*^2^ = 67%, *p* *<* 0.01); non-treatment subgroup: (SMD = 0.08; 95% CI = −0.2 to −0.39; *p* < 0.0001; heterogeneity *I*^2^ = 19%, *p* = 0.29) (Supplementary Material, Figure 7). Egger's test showed that there was no obvious publication bias of statistical significance (intercept −0.03; 95% CI = −2.06 to 2.12; *p* = 0.98) (Supplementary Material, Figure 1).

**Figure 6:**
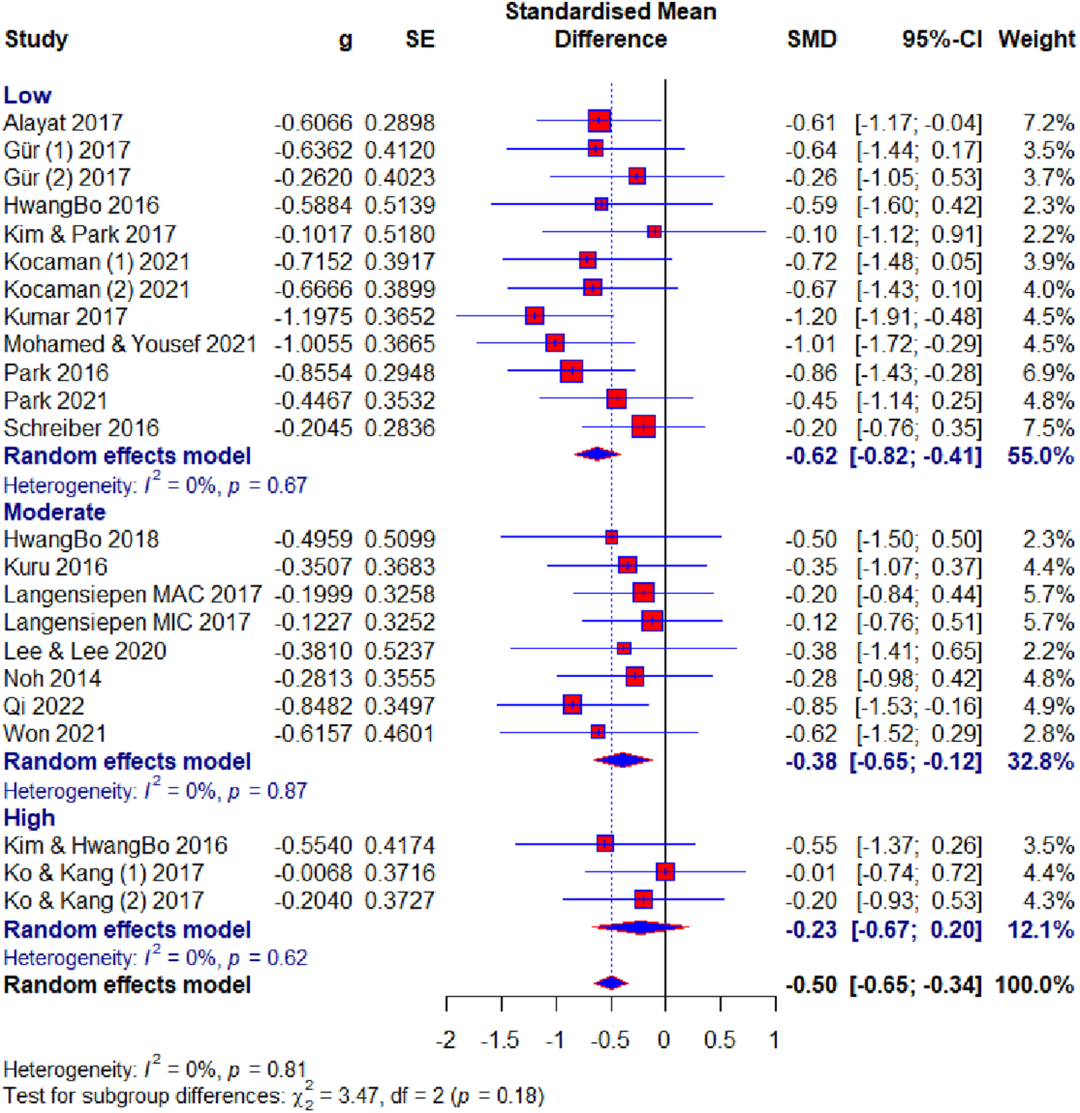
Forest plot— Cobb angle outcome: risk of bias subgroups.

**Figure 7:**
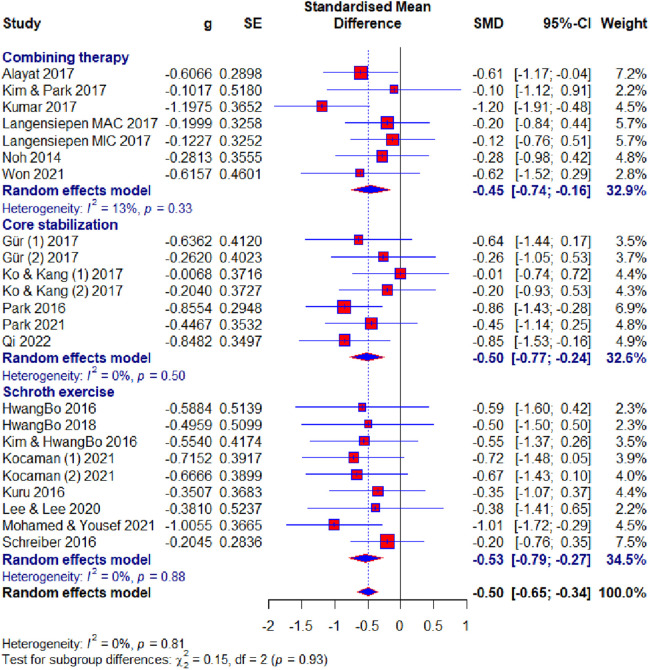
Forest plot—Cobb angle outcome: methods subgroups.

### ATR, FVC, FEV1, and QoL

Four studies used ATR as an outcome. After the analysis, statistical significance was observed (SMD = −0.48; 95% CI = −0.83 to −0.13; *p* = 0.01) and there was no heterogeneity (*I*^2^ = 0%, *p* = 0.62) (Supplementary Material, Figure 2). Three studies used FVC as an outcome. After the analysis, no statistical significance was shown (SMD = 0.58; 95% CI = −0.04 to 1.20; *p* = 0.07) and there was heterogeneity (*I*^2^ = 49%, *p* = 0.14) (Supplementary Material, Figure 3). Three studies used FEV1 as an outcome. After the analysis, statistical significance was shown (SMD = 0.61; 95% CI = 0.19–1.03; *p* = 0.005) and there was no heterogeneity (*I*^2^ = 0%, *p* = 0.53) (Supplementary Material, Figure 4). Four studies used QoL as an outcome. After the analysis, statistical significance was shown (SMD = 1.17; 95% CI = 0.88–1.45; *p* < 0.0001) and there was no heterogeneity (*I*^2^ = 0%, *p* = 0.39) (Supplementary Material, Figure 8).

**Figure 8:**
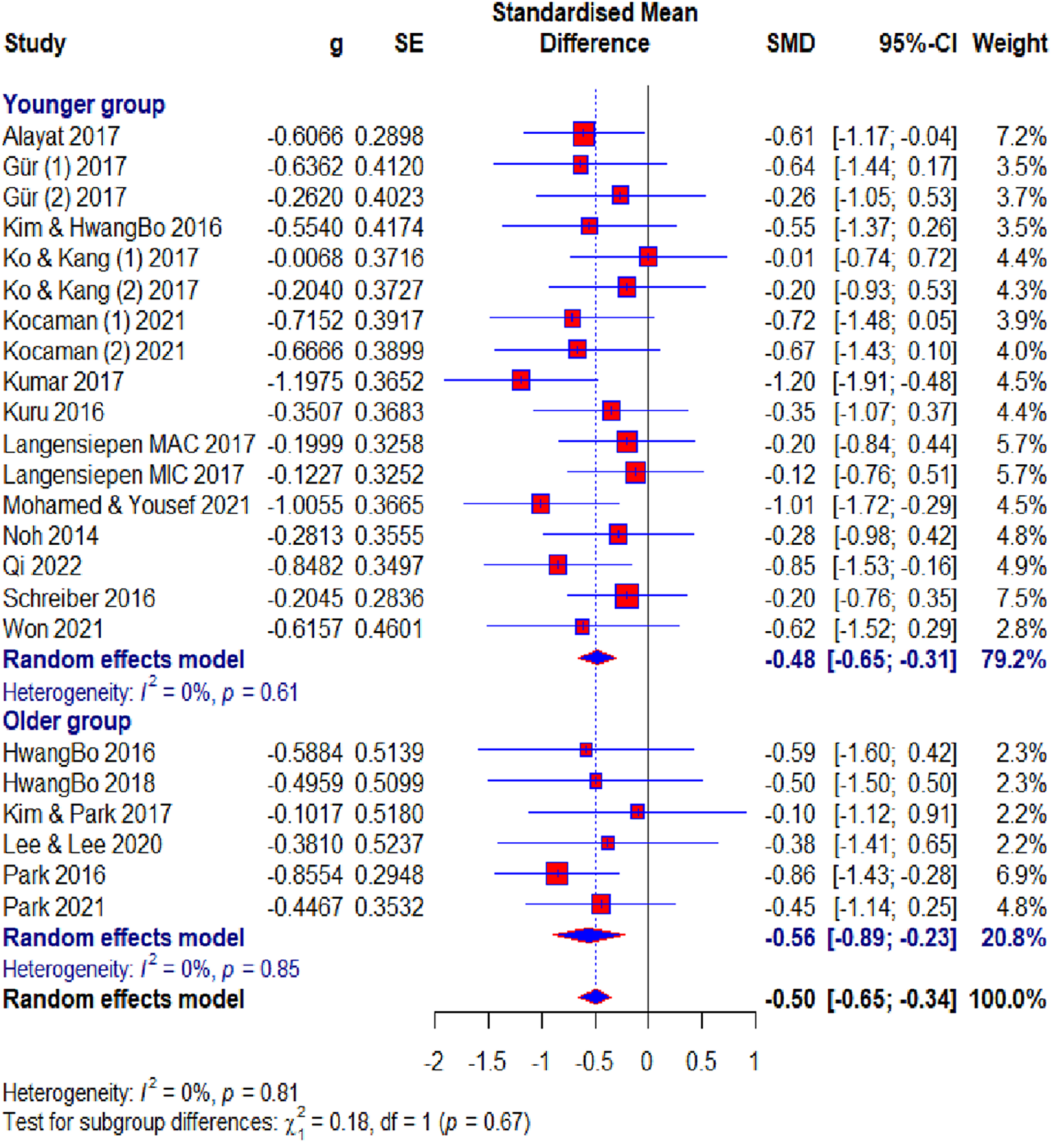
Forest plot—Cobb angle outcome: age subgroups.

## Discussion

**Figure 9:**
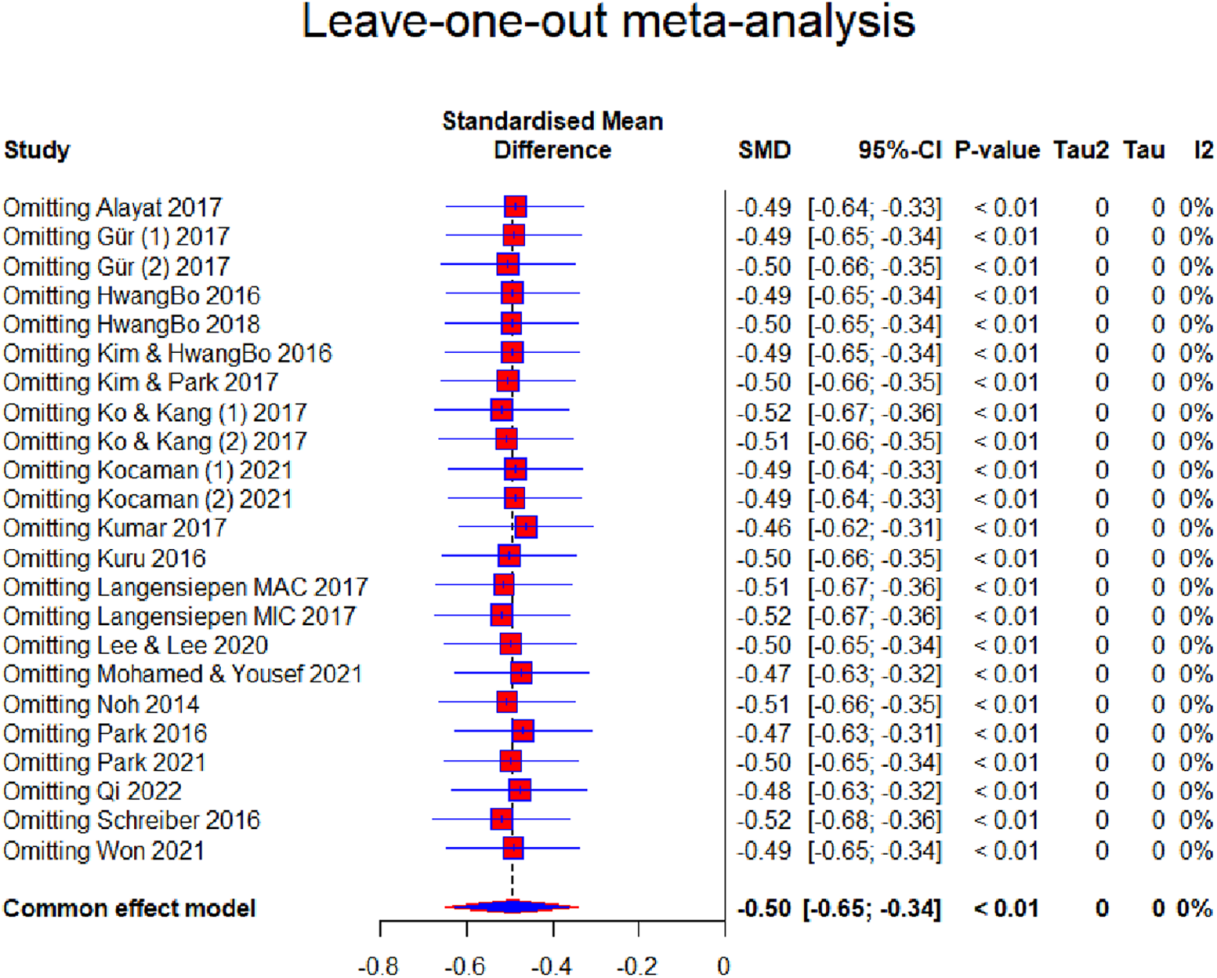
Forest plot—leave-one-out meta-analysis.

This research aimed to use a meta-analysis to determine the effect size of the conservative methods based on exercises on patients of IS. In total, 23 studies, involving 796 subjects, were included in the meta-analysis. The effect size was assessed in five outcomes. The effect size for the Cobb angle outcome was moderate and ATR had an almost moderate effect size. Furthermore, the QoL outcome had a large effect size, the FVC outcome had a moderate effect size, and the FEV1 outcome had a moderate effect size. Sensitivity analysis for the Cobb angle showed that the results range from 0.46 to 0.52 (Figure 9).

**Figure 10:**
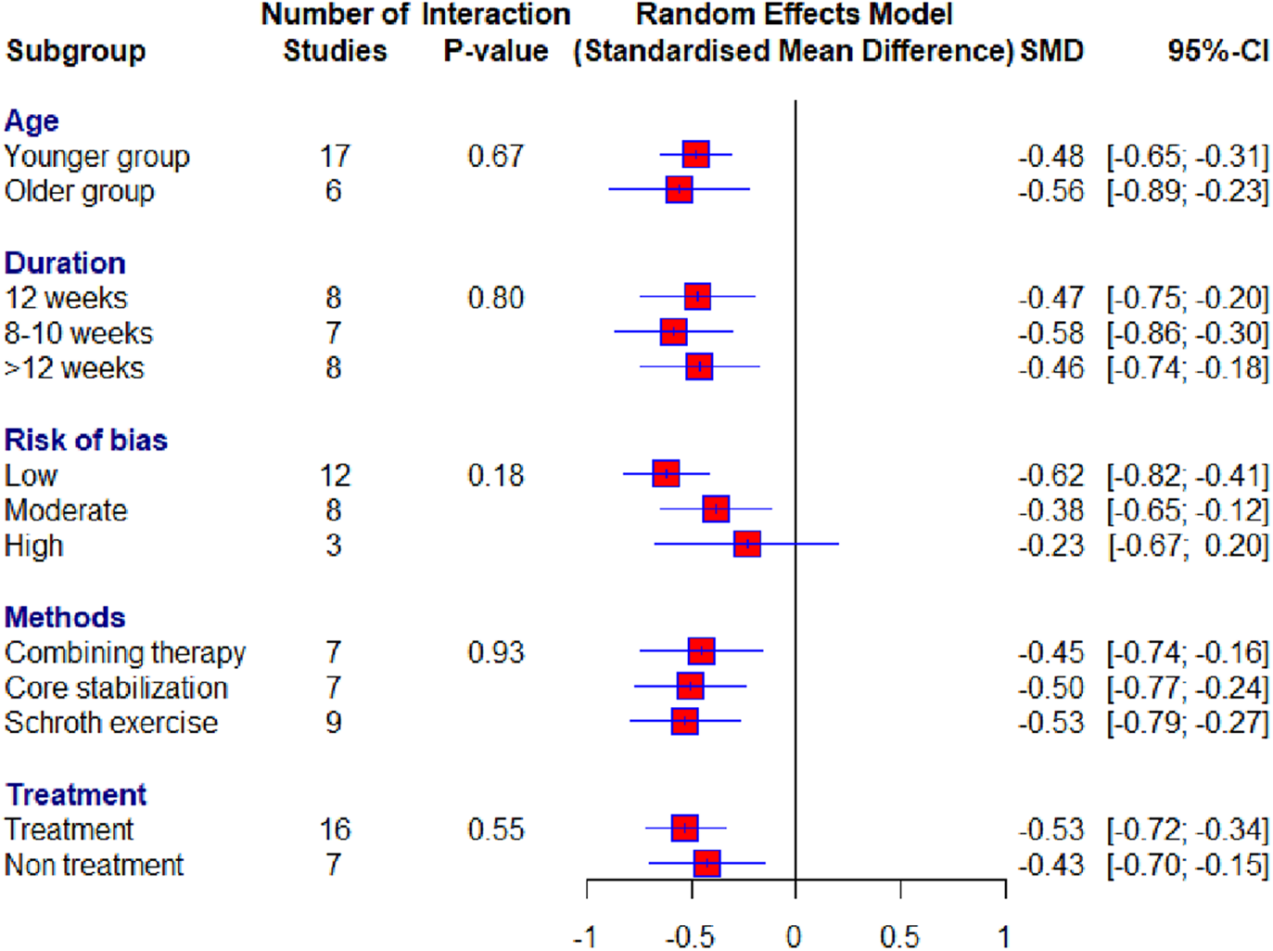
Forest plot—summary of subgroup analyses.

In our meta-analysis, the respondents were diagnosed with IS, and different types of conservative (non-surgical) treatment based on exercises were used as treatment methods. In addition to the Schroth method, which was the most used treatment, a certain number of studies used the core stabilization method, while the rest of the studies used other treatments. For this reason, we classified all other studies into the combined therapy subgroup so that we could compare them with the aforementioned methods, which may represent bias in the decision-making process. Combined therapy led to moderate improvements in Cobb angle outcome. This indicates a synergistic effect when different exercise techniques are combined, which may cater to the diverse needs of individuals with IS. Clinicians should consider combining therapies as a versatile strategy, especially for patients who may benefit from a more comprehensive and multifaceted treatment plan. Core stabilization exercises showed a moderate effect, suggesting they target essential muscular control and spinal alignment, critical for managing scoliosis. In clinical settings, core stabilization exercises should be emphasized, particularly for patients with weakened core muscles or those requiring enhanced postural control. Schroth exercises demonstrated the greatest effect size among the subgroups, highlighting their targeted approach in IS management. Given their strong and consistent efficacy, Schroth exercises should be prioritized as a primary intervention for IS (Figures 7 and 10). Training practitioners in this specialized technique could enhance treatment outcomes. The significant and consistent results across all subgroups underscore the critical role of exercise-based conservative methods in the treatment of IS. Individualized treatment plans can be developed by leveraging the unique strengths of each approach, potentially combining therapies for patients with diverse needs or focusing on Schroth or core stabilization exercises for targeted interventions. These findings advocate for the inclusion of these exercise modalities in standard clinical guidelines and emphasize the importance of further training and resource allocation to implement these strategies effectively.

The majority of respondents were of adolescent age, while some studies had respondents older than 18 years and for these reasons, we performed a subgroup analysis for age. The results suggest that starting exercise-based treatment earlier in life may yield consistent effects. Younger patients might benefit from their greater musculoskeletal plasticity and the potential for spinal growth modification. Despite reduced skeletal growth potential, exercise-based conservative treatments are still highly effective in managing IS in older patients. This suggests that such interventions are beneficial regardless of age, although mechanisms such as improved muscle support and spinal alignment may play a more prominent role in older patients. The slightly larger effect size in the older group may indicate that older patients achieve greater observable improvement, possibly due to better adherence to treatment protocols. The differences between these two populations in the variation in curve progression in spinal flexibility, rigidity, and muscle elasticity would likely lead to opposite results.

The application of the treatment ranged between 6 weeks and 6 months. Subgroup analysis suggests that treatment duration plays a significant role, with the most significant improvements observed in interventions lasting 8–10 weeks. This finding is clinically relevant as it provides practitioners with an optimal time frame for structuring exercise programs. This suggests that intensive, short-term programs may be as effective as longer ones, offering a feasible treatment period for patients and reducing the risk of intervention fatigue. Longer programs also demonstrate moderate effects. Sustained therapy may still be valuable for patients requiring gradual improvements or those with more severe curvatures. The lack of significant subgroup differences indicates that treatment duration (within the examined timeframes) does not drastically alter effectiveness. This provides flexibility for clinicians to tailor programs based on patient availability, preferences, and adherence potential. Low heterogeneity across most subgroups indicates robustness in the findings, which enhances confidence in applying these results to diverse patient populations.

Exercise-based treatments can effectively reduce ATR, a key marker of IS severity, addressing both cosmetic and postural concerns. These outcomes enhance patient satisfaction and indicate the value of incorporating exercises into IS management. The large effect size and statistical significance highlight the positive impact of exercise-based treatments on patients’ quality of life. This includes physical, emotional, and social dimensions, which are often affected by IS. These interventions may serve as holistic strategies to enhance patient wellbeing beyond physical improvements. Our meta-analysis highlights that conservative exercise-based treatments can positively influence respiratory function in patients with idiopathic scoliosis, particularly FEV1, suggesting better expiratory flow and lung efficiency. However, the variable results for FVC indicate a need for further investigation into factors influencing lung capacity outcomes. The lack of statistical significance suggests that the impact of exercise-based interventions on lung capacity is inconsistent. These findings underscore the importance of incorporating pulmonary-focused exercises to optimize respiratory health in scoliosis management. The findings, in addition to all of the above, confirm that conservative exercise-based treatments are beneficial regardless of whether the control group receives alternative treatments or no treatment at all, and justify prioritizing these treatments in clinical practice. Furthermore, the within-group results support the use of conservative exercise-based treatments as an effective evidence-based approach in the management of IS while simultaneously emphasizing the necessity of active interventions. In the 23 studies, we were able to calculate the effects on five outcomes, which represents a good source for drawing conclusions regarding the application of conservative methods based on exercise in the treatment of IS. Patients using conservative methods based on exercise for IS may be encouraged by these results.

Three studies were non-randomized, while the other 20 studies were randomized. Of the randomized trials, none had high risk in more than one item. The most frequent high-risk item was “allocation concealment”. All the non-randomized studies were comparable with sufficient test scores to be included in the study. Heterogeneity in the Cobb angle, ATR, QoL, and FEV1 outcomes was 0% while for the outcome FVC it was 49%, so the results give a true depiction of the effect size. Studies whose results led to heterogeneity were immediately excluded from further analysis. Thus, the results of one study (50), for the ATR outcome, were immediately excluded because analyses in our previous study (15) show that they led to increased heterogeneity. For the Cobb angle outcome, one study (24) also had the effect of increasing heterogeneity, so results from this study for this outcome were excluded.

Studies with a low risk of bias showed the most substantial reduction in scoliosis curvature, emphasizing the importance of high-quality research in determining the true effectiveness of exercise therapy. While less impactful than low-risk studies, moderate-quality evidence also supported the benefits of exercise therapy, suggesting its applicability even in less controlled settings. Studies with a high risk of bias reported the smallest effect size, which may reflect overestimation or underestimation of outcomes due to methodological flaws. Clinicians should critically appraise such evidence before incorporating it into practice. High-quality studies show more substantial benefits, reaffirming the need for rigorous research designs in this field. Despite variations in quality, exercise therapy consistently demonstrates moderate effects in reducing scoliosis curvature, supporting its role as a conservative treatment option.

The search included four databases without language restrictions. Since we have been studying this topic for years, we believe that no study that would meet the conditions for inclusion was left out. The number of studies evaluating the Cobb angle outcome was at a satisfactory level, while the number for ATR, QoL, FVC, and FEV1 outcomes was small, and the results obtained may be limited by this. Of all the included studies, only one study (47) did not present certain results as required for a meta-analysis, but this problem was resolved according to the method proposed by Higgins et al. (38), Furukawa et al. (55), and Hozo et al. (56). In this case, a sensitivity analysis was not performed because these recommendations gave good results.

Compared with previous meta-analyses, our results are closely aligned with those reported by other investigators in terms of Cobb angle (15, 16, 57–59), ATR (15, 16, 58), and QoL (15, 16, 58, 60), reinforcing the established efficacy of these non-surgical approaches for the management of IS. The smaller variations in effect sizes across studies likely reflect differences in the size and design of the included studies, such as the number of participants and the specific treatment protocols used. In particular, our study included a broader range of conservative treatments beyond the Schroth method, including core stabilization and combined therapy, which offers a more comprehensive view of exercise-based approaches. This broader perspective is particularly valuable for clinical practice, as it highlights that several exercise-based methods can produce similar improvements in key outcomes, although the Schroth method may offer a slight advantage. Our meta-analyses on other spinal problems (35, 36) also show the positive effects of applying certain conservative methods based on exercise. In our discussion, we did not compare our results with those of the individual studies included in our analysis because it was methodologically unjustified as they were focused on two different methodologies and the goal of a meta-analysis is to unify the results of many studies.

The main limitation of this study is the inclusion of non-adolescent subjects in the analysis, and we addressed this issue through subgroup analysis. Another limitation of this study is the heterogeneity of treatments used in the combined therapy subgroup. The third limitation of our meta-analysis is the heterogeneity of treatment duration in the included studies, which we attempted to address with three subgroups. Although the pooled and subgroup results suggest a clear homogeneity of the included studies, the results for the FVC outcome showed a heterogeneity of 49%, and this may be a limitation in terms of the true effect size for this outcome. For the outcomes, ATR, QoL, FVC, and FEV1, the number of included studies was relatively small, but only these studies met the inclusion criteria. Of the 23 included studies, 19 (82.6%) used the Cobb angle as an outcome, 4 (17.4%) used ATR and QoL as the outcomes, and 3 (13%) FVC and FEV1 as the outcomes. Thus, a recommendation for future research in the treatment of IS is that it should be conducted in relation to the measurement of these neglected outcomes. Primarily, it should include outcomes that diagnose the pulmonary function of patients with IS. Our results encourage future researchers to do so.

## Conclusion

This study indicates that conservative methods based on exercise overall have positive effects on patients with IS. The effect size ranged from 0.48 to 1.17 for different measured outcomes. In our opinion, our analysis included a sufficient number of studies and had a sufficient number of outcomes. The limitations of this study should be worked on in the future. Our results send an encouraging message primarily to patients that they can use conservative methods based on exercise for IS, and also to physiotherapists and kinesiotherapists who encounter such problems in practice.

## Data Availability

The original contributions presented in the study are included in the article/Supplementary Material, further inquiries can be directed to the corresponding author.
