# Supplementary material for: Treatment of idiopathic scoliosis with conservative methods based on exercises: a systematic review and meta-analysis
Source: Front Sports Act Living. 2024 Dec 23;6:1492241. doi: 10.3389/fspor.2024.1492241 (PMC11700739; doi:10.3389/fspor.2024.1492241)
Supplement: Supplementary file 1 [file Table1.docx]

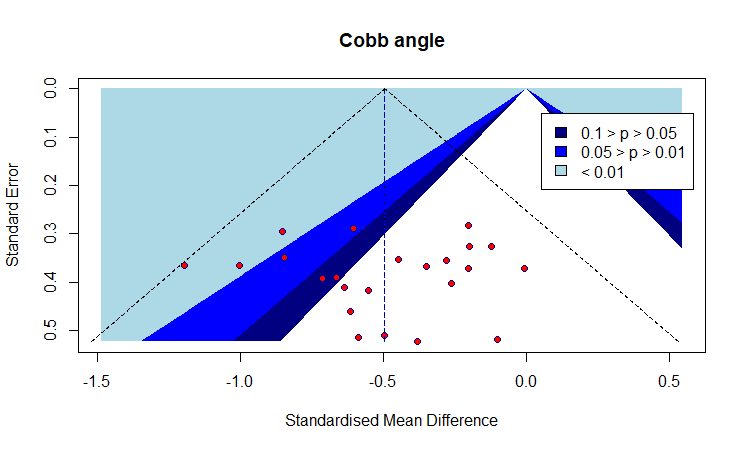


Figure 1. Funnel plot – outcome Cobb angle


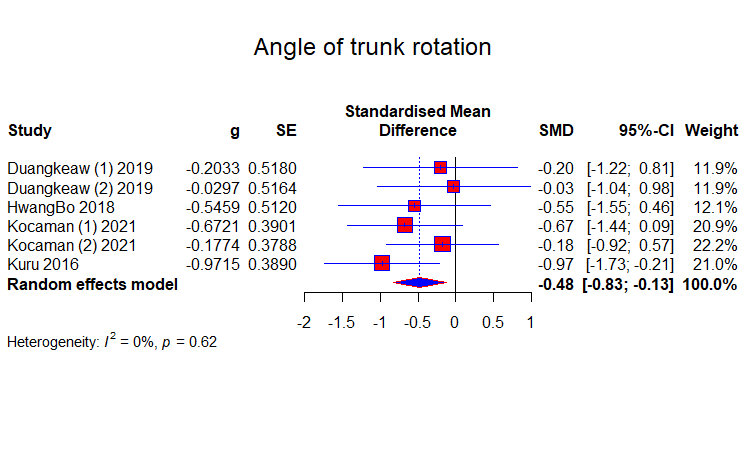


Figure 2. Forest plot – outcome ATR


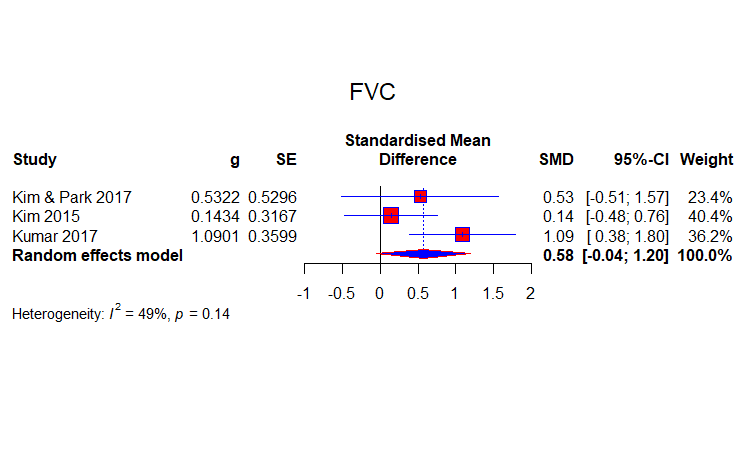


Figure 3. Forest plot – outcome FVC


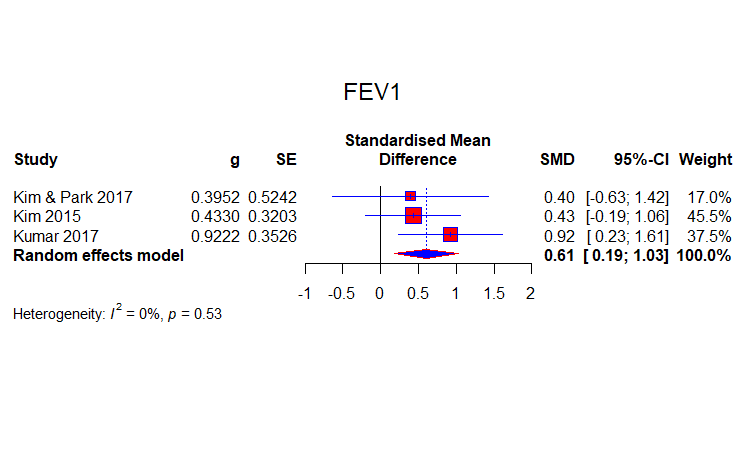


Figure 4. Forest plot – outcome FEV1


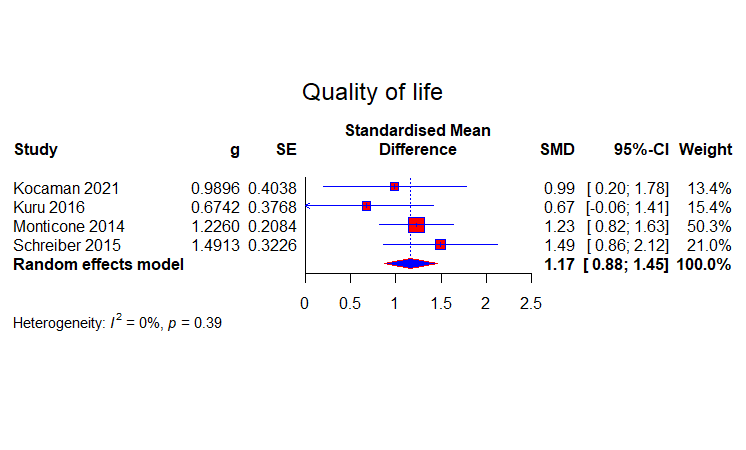


Figure 5. Forest plot – outcome QoL


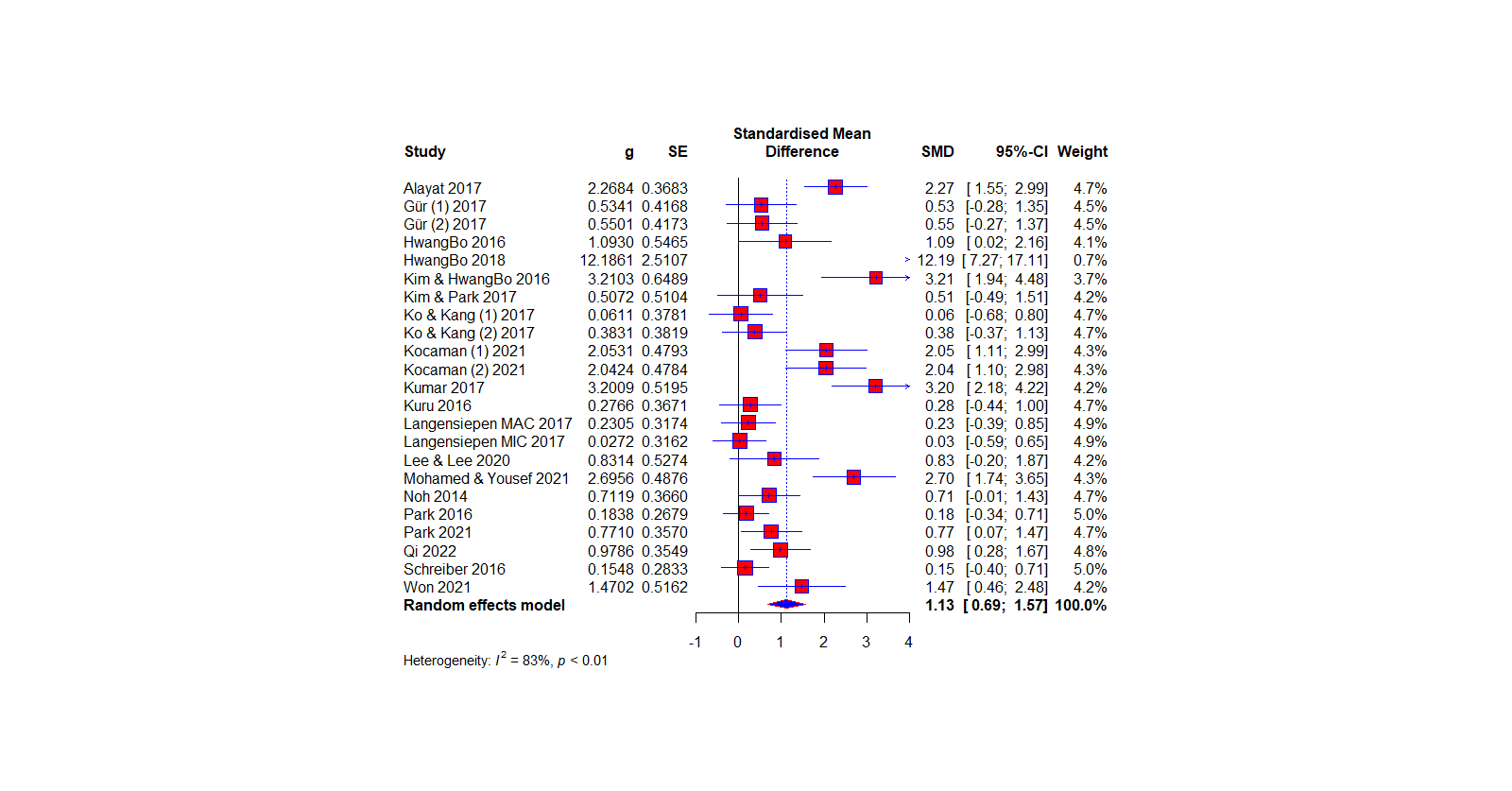


Figure 6. Forest plot – outcome Cobb angle: Experimental group


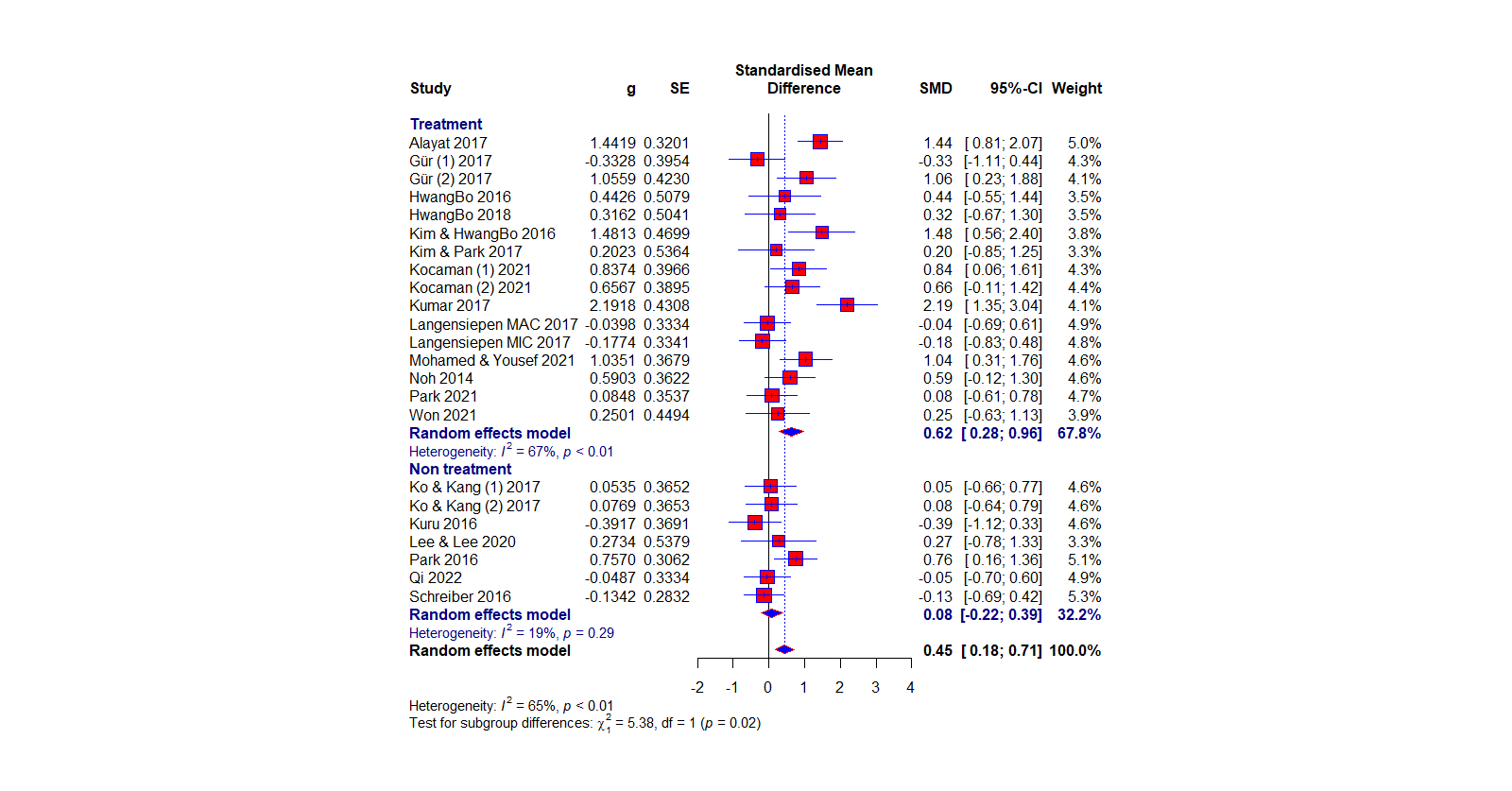


Figure 7. Forest plot – outcome Cobb angle: Control group
